# An Interspecific *Nicotiana* Hybrid as a Useful and Cost-Effective Platform for Production of Animal Vaccines

**DOI:** 10.1371/journal.pone.0035688

**Published:** 2012-04-23

**Authors:** Huai-Yian Ling, Aaron M. Edwards, Michael P. Gantier, Kathleen D. DeBoer, Alan D. Neale, John D. Hamill, Amanda M. Walmsley

**Affiliations:** 1 School of Biological Sciences, Monash University, Clayton, Melbourne, Victoria, Australia; 2 Monash Institute of Medical Research, Centre for Cancer Research, Clayton, Melbourne, Victoria, Australia; 3 Department of Anatomy and Development Biology, Monash University, Clayton, Melbourne, Victoria, Australia; 4 Department of Forest and Ecosystem Science, University of Melbourne, Creswick, Victoria, Australia; Instituto Butantan, Brazil

## Abstract

The use of transgenic plants to produce novel products has great biotechnological potential as the relatively inexpensive inputs of light, water, and nutrients are utilised in return for potentially valuable bioactive metabolites, diagnostic proteins and vaccines. Extensive research is ongoing in this area internationally with the aim of producing plant-made vaccines of importance for both animals and humans. Vaccine purification is generally regarded as being integral to the preparation of safe and effective vaccines for use in humans. However, the use of crude plant extracts for animal immunisation may enable plant-made vaccines to become a cost-effective and efficacious approach to safely immunise large numbers of farm animals against diseases such as avian influenza. Since the technology associated with genetic transformation and large-scale propagation is very well established in *Nicotiana*, the genus has attributes well-suited for the production of plant-made vaccines. However the presence of potentially toxic alkaloids in *Nicotiana* extracts impedes their use as crude vaccine preparations. In the current study we describe a *Nicotiana tabacum* and *N. glauca* hybrid that expresses the HA glycoprotein of influenza A in its leaves but does not synthesize alkaloids. We demonstrate that injection with crude leaf extracts from these interspecific hybrid plants is a safe and effective approach for immunising mice. Moreover, this antigen-producing alkaloid-free, transgenic interspecific hybrid is vigorous, with a high capacity for vegetative shoot regeneration after harvesting. These plants are easily propagated by vegetative cuttings and have the added benefit of not producing viable pollen, thus reducing potential problems associated with bio-containment. Hence, these *Nicotiana* hybrids provide an advantageous production platform for partially purified, plant-made vaccines which may be particularly well suited for use in veterinary immunization programs.

## Introduction

Transgenic plants are gaining acceptance as a platform for the production of affordable recombinant proteins in the pharmaceutical industries [1], [2]. Since the first manuscript reporting plant-made vaccine (PMV) production [3], many studies have demonstrated the value of expressing antigens in plants [4], [5]. Advantages associated with using a plant expression system include the ability to utilize gene splicing to produce multi-antigen vaccines and the decreased risk of product contamination with human or animal pathogens. Plant-made heat stable vaccines can be shipped and stored without refrigeration and have the potential to be produced in edible plant organs and delivered orally without the requirement for recombinant protein purification. Encapsulation within the plant cell wall may increase the oral efficiency of the vaccine and stimulate mucosal and systemic immune responses, and hence may be effective against respiratory infectious diseases [6]. Influenza is a respiratory condition in animals and humans caused by enveloped, segmented, single-stranded, negative sense RNA members of the *Orthomyxoviridae* family of viruses [7]. Avian influenza virus (AIV) can infect a variety of avian and mammalian species including domestic poultry and humans, and poses a serious international pandemic threat [8]. It may be possible to diminish the risk of a pandemic outbreak of AIV by immunising susceptible farm animals against the virus. In fact, the first plant-made vaccine (PMV) to be commercially licensed was a partially purified, injectable poultry vaccine against Newcastle Disease Virus (NDV) [9]. Production of cheap and effective vaccines is particularly important for the developing world where ready access by subsistence farmers to refrigerated, expensive animal vaccines is often extremely limited. As current regulations allow crude or partially purified veterinary vaccine formulations to be administered by injection (http://www.aphis.usda.gov/animal_health/vet_biologics/publications/memo_800_301.pdf), a plant-made vaccine that could be administered without requiring antigen purification may make immunization of large numbers of at risk farm animals economically feasible.

Several studies have reported the successful plant-based production of an AIV surface protein, the haemagglutinin (HA) glycoprotein, using transient transformation in *Nicotiana* [10], [11]. *Agrobacterium*-mediated transient expression of HA in *N. benthamiana* enabled high accumulation (50 mg/kg) of virus-like particles (VLPs) consisting of HA antigen. These plant-made VLPs were purified within three weeks of introduction of DNA constructs into leaf tissues. Mice that were immunised intramuscularly with two doses, each containing 0.5 µg, of the purified H5-VLPs were protected against H5N1 influenza virus challenge [10]. Similarly, a TMV-based, deconstructed viral transient expression system in *N. benthamiana* leaf tissues produced a HA yield of 60 µg/g fresh weight. Following purification, the antigen elicited strong H5-specific immune responses in mice and displayed high haemagglutination inhibition (HI) and virus-neutralizing (VN) antibody titres. The purified plant-made HA also provided full protection to ferrets challenged with the A/Indonesia/05/05 influenza virus [12].

Stably transformed *Nicotiana* species are also well suited for producing plant-made vaccines and offer some advantages over transient production systems. Stable transformation is easily achieved in *Nicotiana* and eliminates the need for repeatedly introducing constructs as well as reducing the potential for batch variation inherent in transient production systems. Furthermore, plants such as *N. tabacum,* grow rapidly and can produce large amounts of leaf biomass per hectare potentially containing high concentrations of antigen (reviewed in [13]). *Nicotiana* species are not food crops and hence the possibility of contaminating the food chain is reduced. However, there are some disadvantages associated with the use of transgenic *N. tabacum* to produce vaccine proteins. *N. tabacum* has the capacity for prolific production of small seeds which are easily distributed and remain viable in the soil for many years. Regulations regarding bio-containment may constrain the use of such a plant as a protein-production system. In addition, leaf tissues of most species in the genus *Nicotiana*, including *N. tabacum*, can contain substantial quantities of toxic pyridine alkaloids, particularly nicotine, nornicotine, anatabine and/or anabasine [14], [15]. The levels of alkaloids increase in leaf tissues of *Nicotiana* following insect attack or physical damage to aerial tissues, such as removal of the inflorescences or vegetative apices [16], [17], [18], [19], . While it is possible that some metabolic components present in crude leaf extracts have synergistic, adjuvant effects when combined with antigenic proteins to improve the immune response [23], [24], [25], the presence of alkaloids in vaccine preparations of crude or partially purified extracts of tobacco leaf tissues may present regulatory hurdles associated with their use in animals. The requirement to purify the vaccine would increase the cost of veterinary vaccine production in transgenic tobacco. Hence, the ability to reduce the seed and alkaloid production capacity of *Nicotiana* would enhance the prospects for utilizing these plants as a vaccine production platform.

Knowledge relating to alkaloid production in *Nicotiana* has increased rapidly in recent years and has facilitated the use of antisense- or RNAi-mediated technology to down regulate key alkaloid biosynthesis genes [26], [27]. Recently we have demonstrated the use of double stranded RNA gene-silencing to reduce or eliminate alkaloid production in leaf tissues of the tree tobacco *Nicotiana glauca,* even when plants were wounded or decapitated [16]. The availability of these transgenic lines provided the opportunity of crossing them with transgenic HA-producing *N. tabacum* to produce an alkaloid-free HA-containing plant whose leaf extracts would be able to induce an antigen-specific immune response in animals without purification. Previous research has indicated that this inter-specific hybrid *Nicotiana* plant would be viable and potentially have the advantage of being self-sterile [28]. To test this idea, DNA constructs utilizing two promoter sequences, the Cassava Vein Mosaic Virus promoter [CsVMV] or the chimeric octapine synthase-mannopine synthase 4OCS-ΔMas promoter [29], and two HA coding sequences (a native or a plant-optimised sequence) were produced. The HA sequence was designed by Dow Agrosciences LLC to induce an immune response against the avian influenza virus. These constructs were transformed into plants of the *aabb* genotype of *N. tabacum* variety LAFC 53 [22], [30]. This LAFC 53 variety (nic1nic2/aabb genotype) was recently shown to contain mutations in alkaloid pathway regulatory genes belonging to the AP2/ERF family of transcription factors [26] and produces less pyridine alkaloid than its near-isogenic parental line of the Nic1Nic2/AABB genotype [22]. The HA antigen-producing ability of transgenic *N. tabacum* variety LAFC 53 plants containing the various constructs was assessed and selected individuals were then crossed to *A622*-RNAi silenced *N. glauca* to obtain the HA-producing, interspecific hybrid. The capacity for pyridine alkaloid synthesis in the interspecific *N. tabacum* X *N. glauca* hybrid was determined and the immunogenicity of the HA crude plant extract was then assessed in mice trials following subcutaneous injection.

## Results

### Characterization of plant-made HA protein

**Figure 1:**
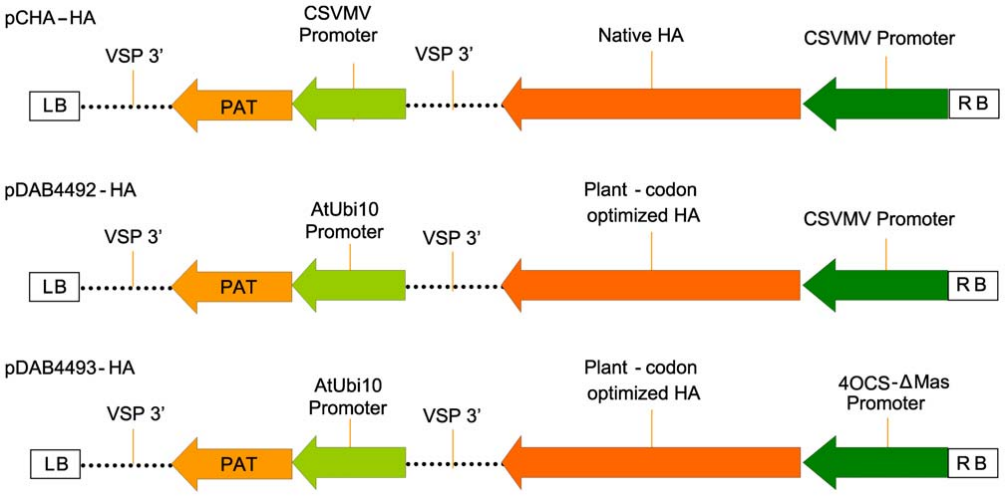
Constructs used to drive expression of HA gene in *N. tabacum* LAFC 53. CsVMV = the cassava vein mosaic virus promoter. 4OCS-ΔMas = a chimeric synthetic promoter. Native HA = the coding region of haemagglutinin from the turkey Wisconsin HA5 AIV strain. Plant-codon optimised HA = the modified haemagglutinin coding sequence based on plant codon usage frequency. LB and RB represent the left and right T-DNA borders respectively. VSP 3′ = the terminator of the soybean vegetative storage protein. PAT = the phosphinothricin acetyl transferase gene.

**Figure 2:**
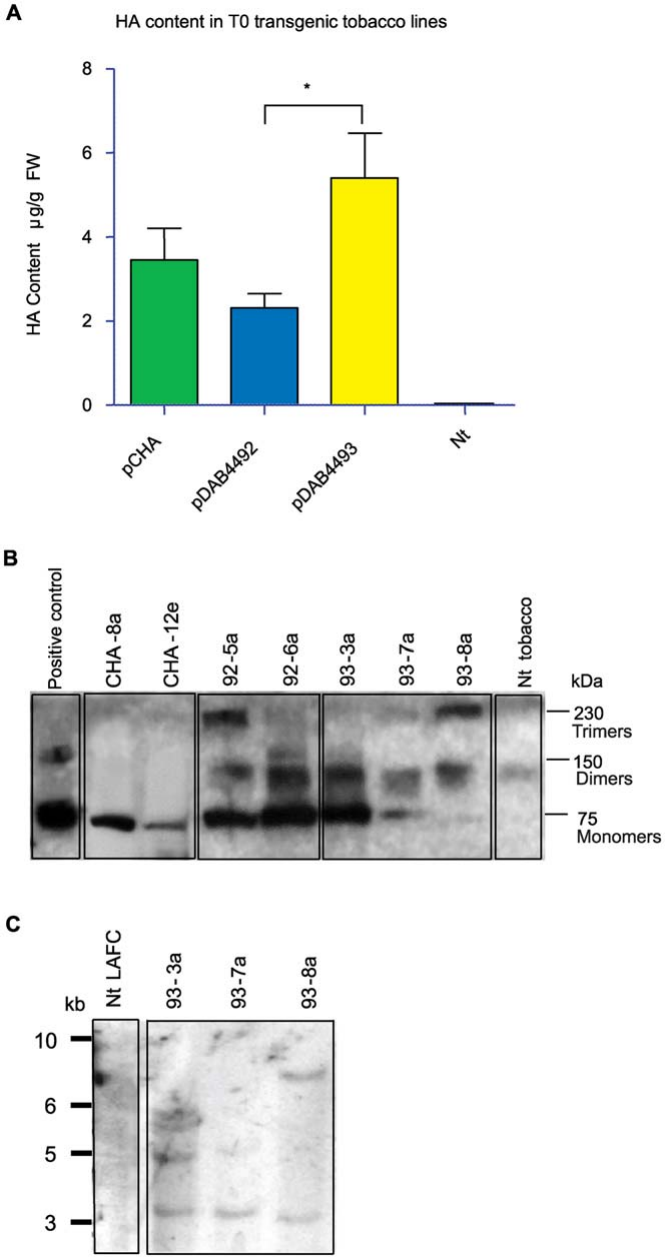
Analysis of T_0_ *N. tabacum* LAFC 53 plant lines transformed with HA constructs. 2a. The mean HA content in *Nt* LAFC-HA plants transgenic for each construct type quantified using ELISA analysis. *Mean levels of HA in plants transgenic for pDAB4493 were significantly different (p<0.05; t-test) to those containing pDAB4492. Nt = non-transgenic *N. tabacum* var. LAFC 53. Error bars represent standard error of mean (SEM) (n = 40 for plants transgenic for pCHA; n = 45 for plants transgenic for pDAB4492; n = 52 for plants transgenic for pDAB4493), FW = fresh weight. 2b. Western analysis of leaf extracts from representative *Nt* LAFC-HA plants. Extracts from plants containing HA constructs were loaded in central lanes as indicated. Purified HA was loaded as a control in the left lane whilst leaf extract from non-transgenic *N. tabacum* var. LAFC 53 was loaded in the right lane. All lanes contained 10 µg total soluble protein (TSP). 2c. Detection of HA transgene in selected elite transgenic lines *of N. tabacum* containing the pDAB4493-HA construct. Southern blot hybridisation of *Hind* III digested genomic DNA isolated from Nt LAFC-HA transgenic plants probed with the HA gene.

To obtain a control sample of plant-made HA protein, *Agrobacterium*-mediated transient expression using a deconstructed viral vector [31] was undertaken in six-week-old *N. benthamiana* plants. Up to 23 µg/g fresh weight (FW) of HA were produced in leaves harvested seven days post infiltration (data not shown). Purification from 600 g of fresh tissue provided 12.6 mg of HA, a yield around 90%. The purified HA was used as a positive control in further analyses. To produce HA in stably transformed plants, transgenic plantlets of *N. tabacum* LAFC 53 were generated containing one of the three expression constructs shown in Figure 1. These *Nt* LAFC-HA plants were screened for their ability to produce HA using capture ELISA. Approximately 80 independent ammonium glufosinate resistant lines for each construct were recovered, and about half were found to accumulate HA protein at levels ≥0.5 µg/g FW. These plants were selected for further analysis. The mean HA content for each construct was calculated from these independent *Nt* LAFC-HA lines from the ELISA analysis, and demonstrated that the *Nt* LAFC-HA plants containing the 4OCS-ΔMas promoter construct, pDAB4493, produced significantly higher HA levels (p<0.05) compared to those produced by plants harbouring the CsVMV promoter construct, pDAB4492 (Figure 2a). There was no significant difference (p≥0.05) between mean HA content of *Nt* LAFC-HA plants transformed with constructs containing either the native or plant codon-optimized coding region (Figure 2a).

All the glufosinate-resistant stable transgenic *Nt* LAFC-HA plants were healthy and showed no obvious phenotypic deviations from normality. From the independent *Nt* LAFC-HA lines, two to three elite plants expressing high HA levels (≥10 µg/g FW) as determined by ELISA were chosen for further analysis.. The presence of the HA gene was confirmed in these plants using PCR (data not shown). Western analysis demonstrated that the monomeric form of the HA protein produced in these elite *Nt* LAFC-HA plants had a molecular weight of approximately 75 kDa, as did the purified HA control (Figure 2b). The HA protein can retain dimeric or trimeric conformation on SDS-PAGE of approximately 150 kDa and 225 kDa respectively, and these were observed in the extracts from several transgenic plants and the HA control. At least two of the highest HA expressing *Nt* LAFC-HA lines, were chosen from lines containing each of the constructs and used for creation of the interspecific hybrid plants.

### Characterization of the interspecific hybrids

We have shown previously that the 35S-*A622*-RNAi construct reduces the high alkaloid levels normally produced by *N. glauca* plants to undetectable levels [16]. To reduce the level of alkaloids produced by the elite *Nt* LAFC-HA plants, they were crossed with *N. glauca A622*-RNAi as described in the methods section. The best performing hybrid line with respect to high HA levels, based on the ELISA results, and the lowest alkaloid content, based on the HPLC data, was selected for further analysis. The parent line of this hybrid, pDAB4493-8a *Nt* LAFC-HA contained two copies of the HA gene (Figure 2c). The same parental pDAB4493-8a *Nt* LAFC-HA line was also crossed with wild-type *N. glauca* to produce an alkaloid-containing hybrid as a control.

**Figure 3:**
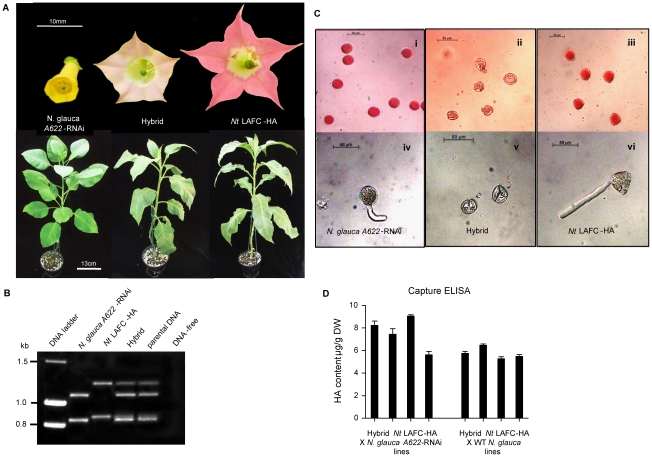
Characterisation of *N. tabacum X N. glauca* hybrids. 3a. Floral and vegetative phenotypes of the hybrid (centre) compared with parentals. All plants in the bottom panel are eight weeks old. 3b. Molecular evidence for the presence of both parental genomes in *N. tabacum X N. glauca* interspecific hybrids. PCR was used to detect species-specific length variability in intron 5 of *Nicotiana QPT* paralogues. *N. tabacum* LAFC 53 generates different sized bands from *N. glauca.* Parental DNA refers to genomic *N. glauca* and *N. tabacum* LAFC 53 mixed *in vitro.* DNA-free refers to PCR performed without template. 3c. Microscopic analysis of pollen. Panels i–iii show pollen after initial staining with acetocarmine. Panels iv–vi shows pollen after incubation in pollen germination medium. Flanking panels show pollen from parental species as indicated. Centre panel shows non-viable hybrid pollen. All scale bars represent 50 µm. 3d. ELISA analysis of HA levels in individual interspecific hybrid plants. Data represents means of triplicate analysis ± SEM. DW = dry weight. All lines are derived from T_o_ parental *Nt* LAFC-HA pDAB4493-8.

Hybrid seeds germinated with high efficiency (>95%). Glufosinate-resistance confirmed the presence of the HA construct. These hybrid plants were vigorous with healthy leaves. The shape and size of the flowers and leaves of the hybrid plants were of intermediate appearance between both parental species (Figure 3a) which is characteristic of interspecific hybrids in the genus *Nicotiana* [32], [33]. To confirm the hybrid nature of these plants, amplification of a molecular marker based on variability in intron length within the *Nicotiana* nuclear gene family encoding quinolinate phospho-ribosyltransferase (*QPT*) (Ryan *et al*., in preparation) was undertaken. The sizes of the fragments generated from PCR analysis of hybrid genomic DNA indicated both *QPT* gene paralogues from the *N. tabacum* and *N. glauca* parents are present in the hybrid plants (Figure 3b). The hybrid plants did not set seed, even after manual application of hybrid pollen, or pollen from either parental species, to stigmata. Further analysis using acetocarmine chromosome staining, revealed that pollen of hybrid plants was empty and shrivelled and incapable of germination, unlike the pollen of both parental species (Figure 3c). This indicates that these transgenic hybrid plants are sterile and is in accord with previous observations made of interspecific hybrids between *N. tabacum* and *N. glauca*, [28], [34].

The presence of the HA antigen was confirmed in the *Nt* LAFC-HA X *N. glauca A622*-RNAi hybrid plants (Figure 3d). There was no significant difference in HA levels in these plants compared to the *Nt* LAFC-HA X wild type *N. glauca* hybrid control plants.

Vegetative shoot regeneration and clonal propagation of the interspecific hybrid Crossing with the tree tobacco *N. glauca* provides the interspecific hybrid with the capacity to grow much larger than the *N. tabacum* parent, affording a substantial increase in biomass production. Under greenhouse conditions, the interspecific hybrid typically reaches a height of about 3 metres before flowering (as opposed to about 1.5 metres for *N. tabacum*) when grown in 0.5 litre pots. Ease of clonal propagation from cuttings and a high capacity for vegetative shoot regeneration are potentially important features in the context of a vaccine production system involving sterile *N. tabacum* X *N. glauca* hybrid plants. To examine clonal propagation, vegetative cuttings from the transgenic hybrid plants were dipped in commercial rooting powder (containing 2 mg/g indolebutryic acid) before transfer to compost. All of these cuttings rapidly developed roots and gave rise to vigorously growing clonal plants (data not shown). This is a feature of both parental species and was therefore expected in the hybrids.

**Figure 4:**
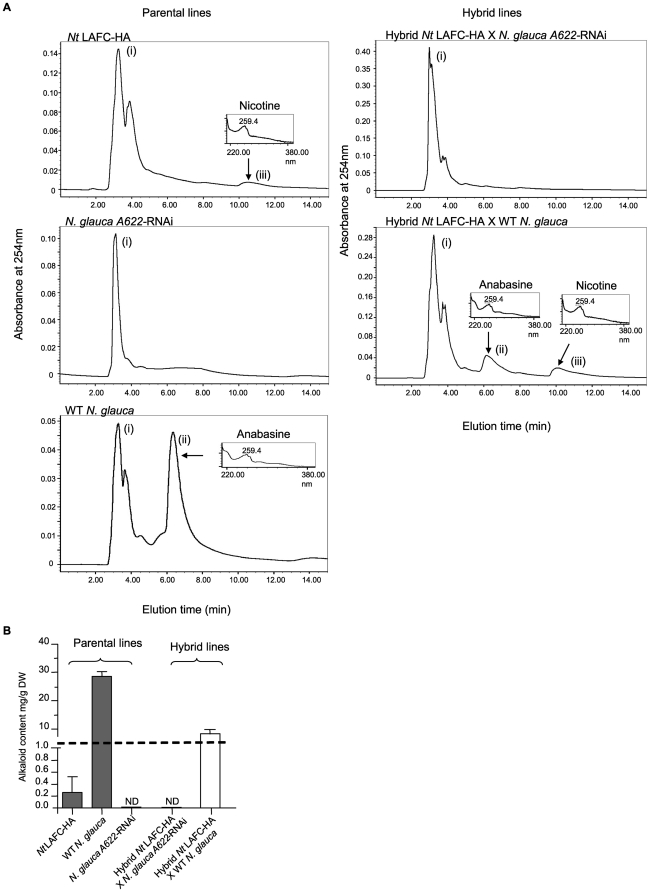
Analysis of interspecific hybrids for alkaloid content. 4a. Chromatograms of the pyridine alkaloid profile in the parental and hybrids lines after removal of plant apices. (i) Nicotinic acid mononucletide was extracted as a background metabolite in the methanol-soluble fraction, (ii) Anabasine profile, (iii) Nicotine profile. 4b. Total pyridine alkaloid levels in vegetative regenerating shoots of parental and WT hybrid controls and HA-containing interspecific hybrid plants. Analysis was undertaken one week after removal of plant apices; graphs represent the mean of three separate plants per treatment (± SEM), ND = None Detected. DW = dry weight.

To test the capacity for vegetative shoot regeneration, the *Nt* LAFC-HA X *N. glauca A622*-RNAi hybrids were decapitated by removal of the top ∼1 cm of their apices. One week after decapitation, vigorously growing vegetative side shoots were observed emerging from the top 4–5 nodes on each decapitated plant. Analysis of these young emerging side shoots showed they contained no nicotine or anabasine, or indeed any other methanol-soluble metabolite with a characteristic pyridine alkaloid spectral absorption profile (Figure 4a). This contrasted with the high levels of alkaloid (mainly anabasine) of almost 10 mg/g dry weight in total, in the corresponding control side shoots of the decapitated *Nt* LAFC-HA X wild type *N. glauca* control hybrid (Figure 4b).

The above results suggest that clonal propagation and repeated mechanical harvesting could be used to scale up the supply of vaccines or other classes of therapeutic proteins in these plants. Such a system may provide an additional advantage as clonally propagated plants may be less susceptible to transgene silencing that can occur during sexual reproduction and hence provide a more predictable level of antigen content in the harvested material [35].

### Immunogenicity and efficacy of plant-made HA in mice

To determine if the HA containing crude plant extract from the interspecific hybrid could invoke an immune responses, the extract was injected subcutaneously into mice. The previously performed ELISA analysis of leaf tissue extracts from both the HA-expressing transgenic hybrids, *Nt* LAFC-HA X wild type *N. glauca,* and *Nt* LAFC-HA X *N. glauca A622*-RNAi (Figure 3d), indicated the presence of 5.4–9.6 µg/g FW of HA. Mice (5–7 individuals per group) were injected subcutaneously with these crude leaf extracts adjusted to contain 5 µg HA antigen. Details of the treatment groups are provided in Table 1. An equivalent amount of purified HA antigen was used in the positive control treatments. In an attempt to minimise any distress in the mice injected with the control tissue extracts that contained pyridine alkaloids, vaccine doses were calculated to contain less than half of the published LD_50_ of nicotine and anabasine [36], [37]. Despite these precautions, some mice injected with samples containing alkaloid exhibited toxic responses. A stage 1 toxic response [38], consisting of a noticeable trauma in the first hour following injection, was observed in one in seven mice of vaccine group E and F following the first injection. A more severe stage 2 response, composed of chronic convulsions lasting up to 2 hours was observed in all seven mice in group E and three of the mice in group F following the second injection. Only one in seven mice in group E and two in seven mice in group F showed a similar response after the third dose. Similar reactions have been observed previously in rats suffering acute nicotine poisoning [38]. No such symptoms were observed in animals receiving either purified HA protein or crude leaf extract from the alkaloid-free transgenic plants.

**Table 1:** Summary of the different experimental groups and candidate vaccine formulations.

Vaccine group*	Origin of vaccine	Vaccine formulations
A	*Nicotiana benthamiana* agroinfiltrated with HA	Purified plant-made HA antigen
B	*N. benthamiana* agroinfiltrated with HA	Purified plant-made HA antigen+alum
C	Hybrid *Nt* LAFC-HA x *N. glauca A622*-RNAi	Crude HA plant extract without alkaloid
cC	Control Hybrid *Nt* LAFC x *N. glauca A622*-RNAi	Control plant extract without alkaloid
D	Hybrid Nt LAFC-HA x *N. glauca A622*-RNAi	Crude HA plant extract without alkaloid+alum
cD	Control Hybrid *Nt* LAFC x *N. glauca A622*-RNAi	Control plant extract without alkaloid+alum
E	*Nt* LAFC-HA	Crude HA plant extract with low alkaloid
cE	Control *Nt* LAFC non transgenic plant	Control non transgenic plant extract with low alkaloid
F	Hybrid *Nt* LAFC-HA x WT *N. glauca*	Crude HA plant extract with normal alkaloid
cF	Control Hybrid *Nt* LAFC x WT *N. glauca*	Control plant extract with normal alkaloid

All HA antigen treatments consisted of 5 µg of HA. 20% alum (aluminium hydroxide) was added as an adjuvant where indicated.

* Lower case “c” refers to the control group for each designated HA treatment group.

**Figure 5:**
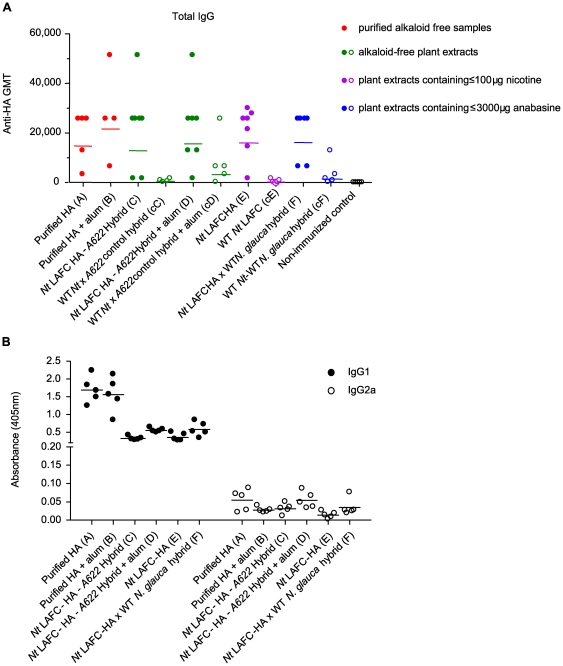
Capacity of HA-containing plant extracts to elicit an immune response. 5a. HA-specific systemic immune response of mice. The horizontal lines represents the geometrical means of HA specific IgG titres at day 28, while the data points represent IgG titres from individual mice. Detailed description of treatments is described in Table 1. Solid dots indicate response to samples containing HA antigen. Open circles indicate responses to samples without HA antigen. A significant difference exits between HA positive samples and control samples (p = 0.0002). 5b. Detection of anti-HA specific IgG isotypes in mice sera on day 28. The horizontal lines represent the geometrical means of IgG isotype titres, while the dots represent IgG isotype titres from individual mice. Detailed description of treatments is described in Table 1.

Serum HA-specific antibody titres in injected mice, as detected by ELISA, peaked on day 28 post-vaccination. By this time, mice immunised with HA treatments possessed significantly higher geometric mean HA-specific serum total IgG titres than their negative-control counter treatments (p = 0.0002) (Figure 5a). No significant difference was observed between HA-specific IgG titres induced by the purified HA or crude HA extracts. While the addition of the adjuvant alum enhanced the response to the purified HA sample, the antigen-specific immunoglobulin (Ig) responses to the HA containing crude extracts were similar, irrespective of the presence or absence of either adjuvant or pyridine alkaloids.

An examination of the IgG isotypes showed high HA-specific IgG1 antibody responses to all HA treatments (Figure 5b), with purified samples eliciting the highest response. The presence of adjuvant did not significantly increase the immune response induced. The IgG2a response was low, leading to high IgG1/IgG2a ratios, ranging from 16.3 to 61.7. The inclusion of adjuvant did not substantially affect these ratios.

**Figure 6:**
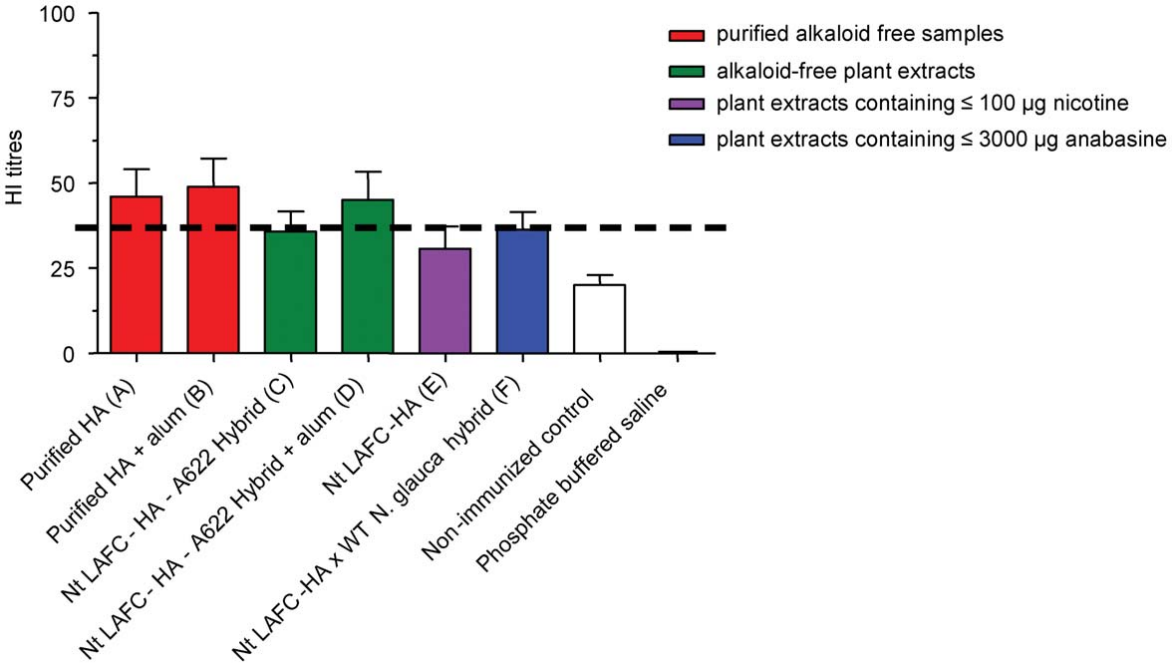
Haemagglutination inhibition (HI) titres of sera from immunized mice. The geometrical means of the HI titres are expressed as reciprocals of the highest dilution of serum that inhibited four haemagglutinin units of virus. Values above the broken line (dilutions of ≥1∶40) indicates the presence of protective HI titres. A significant difference exits between HA samples and the non immunised control (p<0.0001).

Haemagglutinin inhibition (HI) titres using purified split virus (Turkey Wisconsin, H5N9) were measured to confirm the bioactivity of antibodies raised against plant-made HA. Although purified HA treatments (groups A and B) displayed inhibition of viruses at somewhat higher dilutions, there was no significant difference in agglutinating ability between antibodies raised in mice using any of the plant-made vaccine treatments (Figure 6). All transgenic treatments displayed inhibition dilutions that were significantly higher than observed in the negative controls (p<0.0001) (Figure 6).

Overall, these results show that the mouse groups treated with the crude alkaloid-free HA-containing samples developed HA-specific IgG adaptive immune responses and that reducing alkaloid content had no significant impact on the HA specific immune responses induced.

**Figure 7:**
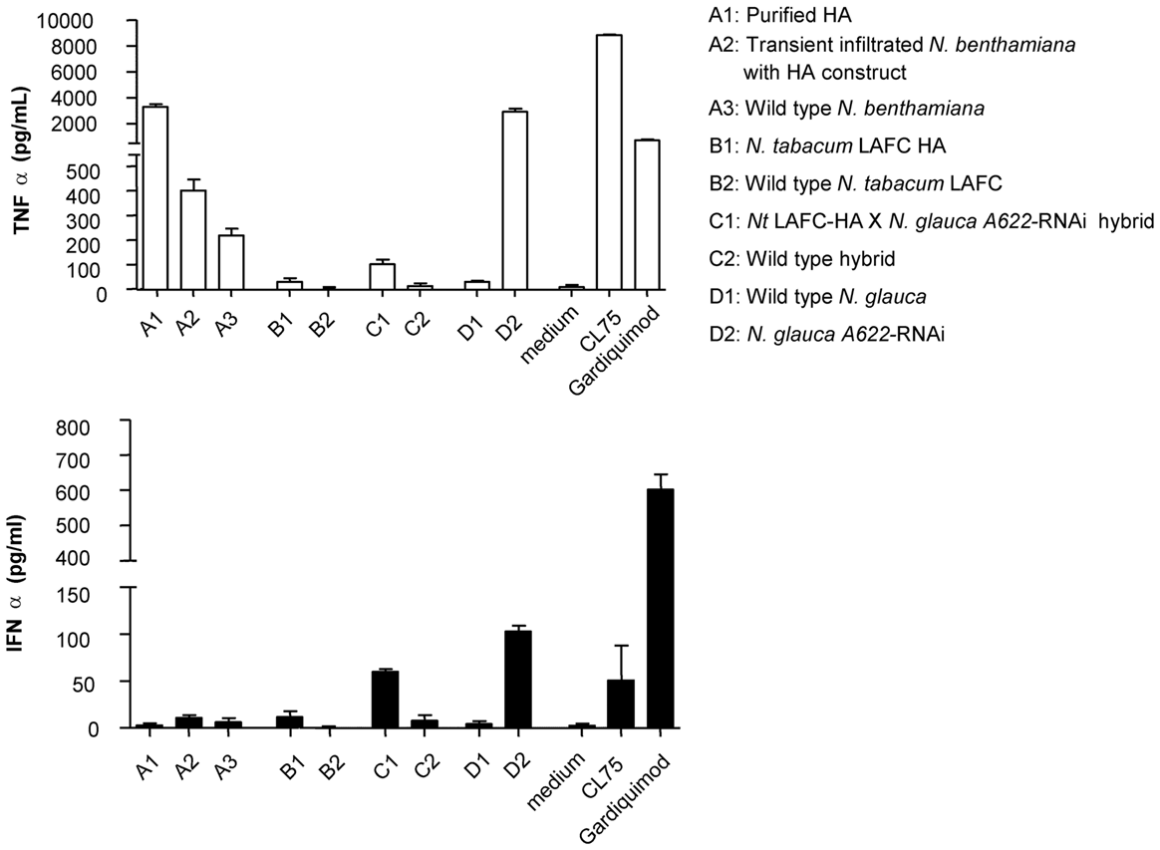
Innate immune response in human peripheral blood mononuclear cells. Cytokine production was measured by specific ELISA, absorbance 750 nm. Data from biological triplicates representative from two independent experiments involving two different blood donors. IFN, interferon; TNF, tumor necrosis factor. Gardiquimod and CL75 were used as controls (see methods).

The innate immune response in human peripheral blood mononuclear cells (PBMCs) was also characterised by monitoring IFNα and TNFα cytokine profiles following overnight incubation (Figure 7). The cytokines released during innate immune responses form a foundation to adaptive immune responses [39]. The cytokine production assay can provide evidence of a possible adjuvant effect associated with the crude plant extract. A high TNFα response, accompanied by a low IFNα response can also indicate the presence of endotoxin. CL75 was used as a control for elevated TNFα production or a response by TLR8, while gardiquimod was used as a control for elevated IFNα production or a response by TLR7. When using human PBMCs, levels of TNFα below 50 pg/mL and IFNα below 50 pg/mL were taken as negligible or negative [40]. The purified HA sample produced very strong TNFα but not IFNα responses. Crude extracts from transiently expressing agroinfiltrated leaves induced a low if not negligible TNFα response although this level was significantly higher than the wild type control (p<0.05). Interestingly, the extract from the control *N. glauca A622-*RNAi lines (not producing HA), resulted in strong TNFα and IFNα responses (Figure 7). The response observed for the control *N. glauca A622-*RNAi extract was mimicked by the *Nt* LAFC-HA X *N. glauca A622*-RNAi hybrid extract, although to a much lesser extent. Our preliminary experiments involving mouse macrophages deficient for TLR2/4 (data not shown) showed that the extract from *N. glauca A622-*RNAi plants induced a strong cytokine response in TLR2/4 deficient cells. Taken together, the results suggest that the innate immune system may be responding to the presence of an unidentified component/s in the *N. glauca A622-*RNAi extract, other than lipopolysaccharide or endotoxins characteristic of gram-negative bacteria.

## Discussion

The potential for a pandemic outbreak has led to classification of H5N1 AIV in List A of the l'Office International des Épizooties (OIE; World Organization for Animal Health). AIV is also recognized as an important constraint to international trade in animals and hence has substantial economic impact [41]. Recently, attention of the scientific community has focused on the development of a plant-made vaccine against avian influenza using transient transformation of *Nicotiana* [10], [11], [12], [42], [43], [44], [45]. These studies used purified proteins to induce an immune response thereby avoiding possible negative health consequences due to the presence of secondary metabolites such as pyridine alkaloids. While stable transgenic *Nicotiana* species have potential as a highly productive, bioreactor species for producing therapeutic and antigenic proteins, allowing rapid vaccine production and ease of scale-up [13], a reduction in the alkaloid metabolites produced in *Nicotiana* may enhance their usefulness. A low-alkaloid, male-sterile tobacco has been used previously to produce recombinant interleukin-10 protein [46], [47]. However, despite the low alkaloid characteristics, the potential usefulness of this female fertile line for large-scale production of antigenic proteins may be limited as most regulatory authorities would be likely to insist on repeated manual removal of inflorescences to ensure adequate environmental bio-containment. Apart from the technical difficulties inherent in ensuring no seed set if such operations were conducted on a large commercial scale, increased alkaloid synthesis is likely to occur in such plants as a result of removal of apices and leaf tissues, albeit at a lower level than in conventional high-alkaloid tobacco varieties [22]. In the present study, we included the use of crude leaf extracts of plants from the low alkaloid variety of *N. tabacum* LAFC 53 stably transformed with HA as a control. This variety of tobacco is capable of producing moderate to high levels of pyridine alkaloid in its roots but generally contains low alkaloid levels in leaves. However, despite having relatively low levels of alkaloid, injection of crude extracts from HA-containing plants of this variety caused some distress in animals consistent with alkaloid toxicity. In contrast the HA-producing interspecific tobacco hybrid which has no detectable alkaloids in the leaf tissues caused no toxicity problems. Moreover, decapitation of this interspecific hybrid encouraged the rapid production of vigorously growing side shoots that were also found to be devoid of alkaloids, indicating that alkaloid levels are likely to remain negligible even in a field situation where tissue damage is likely to occur. The sterile nature, the ease of vegetative reproduction, and the robust side-shoot regenerative capacity of these genetically altered *Nicotiana* hybrids makes them attractive hosts for automated, large scale harvesting of alkaloid-free, vaccine-containing plant material for animal immunisation studies. Whether lack of alkaloids would require such transgenic plants to be protected from increased susceptibility to insect attack if they were grown on a large scale in open plantations is as yet unknown and would be a necessary question to address experimentally if such trials were to proceed.

All plant-made HA treatments in this study produced a substantial increase in HA antibody titre over the corresponding negative control treatments. There was no significant difference between the titres of HA-specific IgG induced by the different transgenic plant-made HA treatments, therefore the sterile and alkaloid-free HA hybrid lines have the same capacity as the purified antigen to induce an HA immune response, without the induction of a toxic effect. The high IgG1/IgG2a ratios induced by plant-made HA treatments indicate a strong Th2 immune response as required for protection against avian influenza [48], however the Th1 response would need to be improved for better protection against the virus. This response may be improved by increasing the HA antigen dose and/or inclusion of a CpG adjuvant [49]. The biological activity of the HA-induced antibodies was verified by testing their ability to inhibit agglutination of purified AIV virus (Turkey, Wisconsin H5N9) to chicken red blood cells. The level of HI titre obtained is generally considered to be biologically significant [50], [51]. Again, there was no significant difference between the neutralising ability of antibodies induced by plant-made antigen from sterile, alkaloid-free hybrid lines and the antibodies induced by purified or alkaloid-containing counterparts.

Immunity in vertebrates is comprised of innate and adaptive immune responses. Once initiated, adaptive immunity is mediated by specific, clonally distributed B and T lymphocytes that undergo 4–7 days of clonal selection and expansion of relevant cell lines [52]. During this delay, infection is controlled by the innate immune response that is mediated through non-clonal recognition receptors that include the toll-like receptor (TLR) pattern recognition receptor family. TLRs are cytoplasmic or transmembrane proteins that can recognize pathogen-derived ligands and initiate complex signal transduction cascades [52]. Each TLR recognizes a diverse range of structurally differing ligands. The best understood of the TLRs is TLR4 that recognizes bacterial lipopolysaccharides (or endotoxins) while others such as TLR 3, 7 or 8 are known to be involved in viral nucleic acid recognition. Engagement of innate immune sensors by pathogens is critical for the proper mounting of an antibody response and is of paramount importance for better vaccine design [53]. We investigated the ability of plant extracts used in this study to induce an innate immune response, and in particular, to determine if possible endotoxin contaminations were present. The very strong TNFα but not IFNα responses observed in our purified plant extract are the hallmark of endotoxin contamination. Since this was not seen in the crude transiently produced HA extracts, we believe contamination of the HA sample occurred some time during the purification process. The strong response for both TNFα and IFNα that was induced by the *A622-*RNAi silenced *N. glauca* extract, and to a lesser extent by the *A622-*RNAi hybrid extract, indicates the presence of an inducer of the innate immune response independent of an endotoxin contaminant in these plant extracts. The induction may have been due to ssRNA present in the RNAi plants, but these products are unlikely to be stable enough to be endocytosed and activate endosomal TLR7 and TLR8 responses. In *Nicotiana*, the alkaloid and polyamine pathways share common precursors. The blocking of the pyrimidine alkaloid pathway due to the absence of A622 activity is likely to result in a build up of intermediate metabolites and/or increase in the flux of precursors through the polyamine pathway. Future work will investigate the nature of the innate immunity inducer/s present in these extracts. Since polyamines have previously been reported in the literature to induce innate immune responses, these are thought to be likely candidates [54], [55]. It should be noted that although previous studies have observed that innate immune responses act as a foundation to enhance adaptive immune responses [39], no substantial difference was observed between the adaptive immune response resulting from crude HA extracts of the *A622*-RNAi hybrid lines, that contain the putative inducer/s of the innate immune system and the HA extracts from non- *A622-*RNAi plants (purified HA extracts, and extracts from non-hybrid/WT hybrid lines). This may be due to a threshold innate immune response not being reached by the hybrid line which evoked a lower response than the *N. glauca A622*-RNAi line (Figure 7), or the lack of conservation of the innate immune sensor(s) involved between human and mouse. Nevertheless, the results indicate the potential for engineering further enhancement of these hybrid lines for veterinary vaccine production purposes, with potential self-adjuvanting properties.

In conclusion, our studies indicate the efficacy of HA-containing, self-sterile, interspecific *Nicotiana* hybrids for production of plant-made influenza vaccines. Elimination of pyridine alkaloids in transgenic hybrid plants reduced toxicity of the crude antigen extracts without adversely affecting plant growth and productivity or the capacity of the plant extract to illicit an immune response. Such alkaloid-free *N. tabacum* X *N. glauca A622*-RNAi interspecific transgenic hybrids therefore have potential as a model vaccine bioreactor system for production of plant-made vaccines against influenza virus and possibly other infectious agents. In particular, the use of such a system for animal immunization studies has potential to improve efficiency of bio-containment procedures and reduce the need for vaccine purification which could substantially diminish the cost of downstream processing.

Lastly, one of the major factors with administering antigens in plant extracts is ensuring consistency in the dosage levels required for effective immunization. The ability to use large-scale clonally-propagated plant material for antigen production has the potential to provide consistent levels of antigen in the plant extract and hence address this problem.

## Materials and Methods

### Production of HA in plants

The HA glycoprotein was produced in *Nicotiana* leaf tissues either by transient transfection with a viral-based vector system (MagnICON) or through stable *Agrobacterium*-based genetic transformation using a binary vector. A combination of native or plant-codon optimized coding regions and the constitutive promoters: cassava vein mosaic virus (CsVMV) promoter or synthetic 4OCS-ΔMas promoter was assessed for the capacity to produce the HA antigen. The pCHA, pDAB4492 and pDAB4493 binary plasmids are proprietary plant expression vectors belonging to Dow AgroSciences LLC. The pCHA and pDAB4492 vectors contain the CsVMV promoter whilst pDAB4493 contains the 4OCS-ΔMas promoter. Both pDAB4492 and pDAB4493 contain the plant-optimized coding region of the HA gene (Figure 1). All plasmids were cultured in *Escherichia coli* DH5α and electroporated into *Agrobacterium tumefaciens* LBA4404 [56]. For preparation of purified HA, the plant codon-optimised HA coding sequence was amplified from the construct pDAB4493, using primers with flanking *Nco*I and *Bam*HI sites, and ligated into magnICON plasmid pICH11599 (Icon Genetics) to produce the pICH-HA sub-clone. *Nicotiana benthamiana* leaves transiently expressing HA were produced using *A. tumefaciens* strain *GV3101* transformed with pICH-HA and co-transformed with the cytoplasm-targeting and integrase modules of the MagnICON system (Icon Genetics GmbH, Germany) [31], [57], [58]. A vacuum infiltration device was used for this process. Plants were inverted so that their aerial portion was submerged in 3 litres of *A. tumefaciens,* OD_600_ of 0.5 before being inserted into the vacuum infiltration device. A vacuum was pulled to 25 mm Hg for 1 min, then quickly released. The plants were returned to the greenhouse and the leaves harvested ten days post-infiltration (dpi) and stored at −80°C until processing.

### Stable plant transformation

Transgenic plants of *Nicotiana tabacum* LAFC 53 (a low-alkaloid variety of *aabb* genotype) [22], [30], containing expression constructs from pCHA, pDAB4492 or pDAB4493 binary vectors, were obtained using a leaf disc transformation procedure as described previously [16]. Independently transformed lines were selected on medium containing 100 mg/L ammonium glufosinate and transferred individually to pots of soil when root systems were well developed. Plants were screened for HA production in leaf tissues when approximately 10 cm in height using an enzyme-linked immunosorbant assay (ELISA). Plants expressing high levels of HA antigen (*Nt* LAFC-HA) were used in subsequent experiments.

### Genomic DNA extraction for detection of the HA gene in transgenic plants

*Nt* LAFC-HA plants expressing high HA levels were selected for further molecular characterisation. Genomic DNA was extracted from 100 mg fresh leaves of T_0_ transgenic lines. Leaf material was collected and snap frozen in liquid nitrogen before being crushed to a fine powder with two 3 mm tungsten carbide beads for 1 min at a frequency of 28/s in a bench-top Qiagen Mixer Mill. Genomic DNA was then prepared using a standard CTAB extraction protocol [59]. Genomic DNA (∼0.5 µg) plants was used as template in PCR analysis using transgene-specific primers; HAF237 (5′-GGSAACCCAATGTGTGATGAG-3′) and HAR1071 (5′-CCATCCATCWAC CATKCCTTGCC-3′) with an annealing temperature of 52°C and a total of 30 cycles. An amplicon of 854 bp was anticipated when using DNA from HA-positive transgenic plants. Southern transfer [60] was performed using 15 µg of DNA from selected elite transgenic *Nt* LAFC-HA plants following digestion with *Hind* III for both pDAB4492-HA and pDAB4493 constructs and with *Stu* I for pCHA-HA. The HA PCR amplicon of 854 bp noted above was labelled by incorporation of digoxigenin (DIG)-labelled dCTP using a PCR DIG Probe Synthesis kit (Boehringer Mannheim, Germany). Probing and visualization was performed using Roche Applied Science DIG wash and block buffer set and a DIG Luminescent Detection Kit. Hybridising bands were detected by exposure of labelled membranes to X-ray film for 2–5 min.

### Purification of reference HA protein

For large scale production of purified plant-made HA, infiltrated leaf samples were homogenized in protein extraction buffer (200 mM sodium borate pH 9, 5 mM EDTA pH 8, 20 mM DTT & 2.0% (w/v) sodium deoxycholate) and filtered to removed cell debris. The lysate was centrifuged at 26,000 g for 30 min and filtered through Whatmann filter paper number 1. Protein was precipitated by adding 1/3 volume of cold ethanol (4°C) and stirring overnight at 4°C, followed by centrifugation at 14,000 g for 45 min. The pellet was resuspended in 600 mL of buffer (50 mM Tris pH 8, 5 mM DTT & 2.0% (w/v) Tween 20) for 1 hour at 4°C followed by centrifugation at 26,000 g for 30 min. The supernatant was extracted with an equal volume of diethyl ether twice and the aqueous phase was degassed and diluted into an equal volume of column buffer contained a final concentration of 0.5% W/V Tween 20, 20 mM Tris-HCl pH 8, 150 mM NaCl, 0.5% (w/v) CHAPS, 1 mM CaCl_2_, 1 mM MgCl_2_ and 1 mM MnCl_2_.

The soluble protein was purified in a two-step purification procedure by Green Chemistry Laboratories, Monash University, Australia using a concanavlin A column and the anion exchange resin Capto Q (GE healthcare). The soluble protein was loaded into a 20 mL conA column. The column was eluted with 20 mM Tris-HCl pH 8, 1 mM EDTA pH 8, 0.1% (w/v) CHAPS, 1 M NaCl buffer and fractions containing HA were pooled and dialyzed (10,000 MWCO Snake Skin, Pierce) against 20 mM Tris pH 8, 1 mM EDTA pH 8 and 0.1% CHAPS at 4°C. The dialyzate was loaded onto the anion exchange column and eluted with 20 mM Tris-HCl pH 8, 1 mM EDTA pH 8, 0.1% (w/v) CHAPS, 1 M NaCl buffer. The purity and quantification of plant–made HA was determined by HPLC-FLD amino acid residue analysis and SDS-PAGE.

### Crude protein extraction

Crude protein was extracted by homogenising leaf material in ice-cold extraction buffer [50 mM sodium phosphate, pH 6.6, 100 mM NaCl, 1 mM EDTA, 0.1% Triton X-100, 1 mM PMSF and Roche EDTA-free Complete Protease Inhibitor Cocktail tablet] with two 3 mm tungsten carbide beads for 1 min at a frequency of 28/s in a bench-top Qiagen Mixer Mill. Insoluble material was removed by centrifugation at 13,000 rpm at 4°C for 5 min. HA content was quantified by capture ELISA.

### HA-specific capture ELISA

Briefly, 96-well titre plates were plated with 50 µL per well of diluted (1∶10,000) goat-anti HA polyclonal antibody in phosphate buffered saline with 0.05% Tween 20 (PBST). The plates were sealed and incubated overnight at room temperature. All subsequent incubations were performed at room temperature and the plates were washed in PBST three times after each incubation step. Plates were blocked with 5% dry milk (5% PBSTM) for 90 min. A volume of 200 µL of crude plant extracts or standard was added per well to the first row of the plate. Samples and standards were serially diluted two-fold down the plate using PBS and the plates were incubation for one hour. A monoclonal mouse anti-HA antibody in PBS (100 µL of 1∶2000 dilution) was added to each well and incubated for an hour. The diluted goat anti-mouse IgG conjugated with horseradish phosphatase (Sigma) (1∶10000 dilution in PBS) was added to the plates and further incubated for an hour. Detection was performed using TMB Peroxidase EIA Substrate kit (Biorad) according to the manufacturer's directions. The amount of HA expressed in the leaf was calculated by reference to a standard curve constructed using purified HA (Benchmark Biolabs). Quantified ELISA data was converted to microgram of HA per gram of fresh weight. Plant lines with HA accumulation higher than 0.5 µg/g in fresh leaves were chosen for further analysis.

### SDS-PAGE and immunoblot analysis

Crude protein extracts from transgenic and wild type *N. tabacum* plants were resolved by SDS-PAGE followed by western analysis. Equal volumes of extracts were loaded on precast 10% polyacrylamide gels (BioRad). 12 ng of purified plant-made HA was used as the positive control. The separated proteins were then transferred to a PVDF membrane (hybond-N, Amersham) before being blocked with filtered 0.1% skim milk in PBS with 0.1% Tween-20 for 16 hours at 4°C on a rocking platform. The membrane was processed with Snap i.d (Millipore) according to manufacturer's instructions. The primary antibody used was a 1 in 2000 dilution of mouse anti-plant HA mAb in blocking buffer (as described above). The secondary antibody was goat anti-mouse IgG conjugated with horseradish phosphatase (HRP) (Sigma) (1∶10,000 dilution in blocking buffer). Detection of reactive protein bands was performed using chemiluminescent substrate (ECL kit) (Amersham) according to the manufacturer's instructions.

### Creation and analysis of transgenic interspecific hybrids

Hybridization between parental genotypes was performed with plants grown in insect-free P2 greenhouse conditions with selected T_0_ plants of HA–positive *N. tabacum* LAFC 53 plants serving as female. Seed pods developed rapidly and several hundred fully formed seeds were harvested approximately 3 weeks after fertilization. Hybrid seed were selected for ammonium glufosinate and kanamycin resistance to ensure the presence of both the HA and the *A622*-RNAi constructs. Growth of plants in hydroponics and alkaloid analysis of vegetative tissues was undertaken as described previously [16], [22], [61]. Pollen viability in hybrid plants and parental lines was estimated by incubating freshly dehisced anthers in a few drop of 2% w/v acetocarmine solution and determining the percentage of stained grains following microscopic observation. Typically, viable pollen is deeply stained and rounded whilst non-viable grains appear shrivelled and lightly stained [62]. The ability of fresh pollen grains to germinate in vitro was also assessed using germination medium [63]. Observations were made after several hours of incubation in medium at 25°C and pollen tube growth was determined under light microscopy Axioskop (Zeiss). Photographs were taken using a Zeiss Axiocam digital camera using Axio Vision software.

### Immunisation of mice with HA antigen

#### Inoculum preparation

Transgenic and wild type freeze dried leaf powder was resuspended in PBS and centrifuged at 12,000 rpm in a Sorvall centrifuge on the immunization day. 20% alum (aluminium hydroxide - Sigma) was added to the designated extracts (Table 1) and incubated for 30 min on ice before dose delivery. The HA vaccines contained 5 µg of purified HA (without alkaloid) or 5 µg of HA in crude leaf extract (containing zero, low or normal levels of alkaloid). For crude leaf extract injection, doses containing ≤50% LD50 of each alkaloid (∼100 µg nicotine; ∼3000 µg anabasine) [36], [37] were delivered. An equivalent amount of alkaloid was injected in the control wild type crude leaf extract treatment. Mice were maintained under conditions approved by the Animal Ethics Committee, School of Biological Sciences, Monash University. Female C57BL/6J mice, six to eight-weeks-old were used to test the immunogenicity of the plant-made HA. Mice were housed in individual cages provisioned with water and standard food and monitored daily for health and condition. Mice were acclimatised for four days during which time they were randomly assigned to ten groups comprising five to seven mice each (Table 1). Groups of mice were subcutaneously injected with 5 µg of purified HA or non-purified plant-made HA leaf extract (with or without 20% of aluminium hydroxide as adjuvant) on days 0, 7 and 14 in treatments as listed in Table 1. After inoculation, mice were observed for any post-immunization response before being returned to the animal house. Serum samples were collected through tail tipping on days −4, 8, 15 and cardiac puncture after CO_2_ inhalation on day 28. Blood samples were allowed to clot at room temperature for one hour before incubation at 4°C overnight. The samples were then centrifuged at 4°C for 10 min at 2500 rpm and the serum (top layer) removed to a new centrifuge tube and stored at −20°C.

### Analysis of the immunogenicity of plant-made HA in mice

Endpoint titre ELISA was performed to determine the anti HA-specific IgG response in mouse sera. High-binding 96-well ELISA plates were coated with 50 µL of HA (1 µg/mL) in carbonate/bicarbonate coating buffer (15 mM NaCO_3_, 35 mM NaHCO_3_, 0.02% NaN3, pH9.6). The plate was placed at room temperature for one hour then further incubated at 4°C overnight. All subsequent incubations were performed at 37°C and the plates were washed in PBST three times after each incubation step. Blocking buffer (5% skim milk in PBS with 0.05% Tween 20) was added to the plate and incubated for 90 min. Serum samples were diluted in 1∶100 in sample buffer (1% skim milk in PBS with 0.05% Tween 20) and added to the first row of the plate, followed by a two-fold serial dilution in PBS down the plate and incubated for 2 hours. The diluted goat anti-mouse IgG conjugated with horseradish phosphatase (Sigma) (1∶5000 dilution in sample buffer) was added to the plate and further incubated for an hour. Detection was performed using TMB Peroxidase EIA Substrate kit (Biorad) according to the manufacturer's directions.

Rabbit anti-mouse class and subclass antibodies from mouse monoAB ID kit (HRP) (Invitrogen) were used for isotyping mouse immunoglobulins (IgG1 and IgG2a,) in sera samples as per the manufacturer's instructions. Absorbance was read at 405 nm using a ThermoMax Microplate reader. Haemagglutination inhibition (HI) assays were carried out as described [64] with some modifications. Briefly, sera from immunized mice were diluted at 1∶10 and heat inactivated in PBS at 56°C for 30 min. Chicken red blood cells (CRBC) in Alsever's solutions (kindly provided by Dr Lori Brown, Melbourne University) were washed three times in PBS and adjusted to the final concentration of 1% in PBS. The purified split AIV virus (H5N9 Dow AgroSciences, Indianapolis, USA) was diluted to four haemagglutinin units (HAU) in PBS as standard antigen. In round-bottomed polystyrene 96-well microtitre plates, the diluted heat-inactivated mice sera were added to the first well and two-fold serially diluted down to the final well of the plate, then 4HAU of virus (1∶1000) was added to all the wells except the CRBC control wells. Plates were incubated at room temperature for 30 min, and 1% CRBC in PBS was added to all wells and further incubated for 30 min at room temperature. Plates were then tilted at about 60° and wells were observed for agglutination. The HI titre was defined as the reciprocal of the greatest dilution of serum causing complete inhibition of agglutination by 4 HAU of antigen.

### Detection of cytokines production

Transgenic and wild type freeze dried leaf powder was resuspended in PBS and centrifuged at 12,000 rpm. Cleared supernatant was used for the analysis as described in [40]. Freshly isolated peripheral blood mononuclear cells (PBMCs) from healthy blood donors were plated in 150 uL RPMI medium supplemented with 5% fetal calf serum and incubated for 4 hours at 37°C (150–200,000 cells per well of a 96 well plate). 50 uL of plant extract were added to each well and incubated for 18 hours. Supernatants were subsequently collected and IFNα and TNFα levels quantified using reported procedures with specific ELISA assays [40]. CL75 also known as 3M-002 (human TLR8 agonist and mouse TLR7 agonist, Thiazoquinoline) and Gardiquimod (human TLR7 agonist, imidazoquinoline), were purchased from Invivogen (San Diego, CA) and used at a final concentration of 1 µg/mL (3M-002 and Gardiquimod).

### Data transformation and statistical analysis

The starting point for the cut-off dilution determining a “no response” was established by determining the first dilution point of each pre-bleed sample where readings plateaued to a slope of less than 0.05. The endpoint titre was estimated as the reciprocal of the highest dilution of the sera that had ≥0.1 OD_450_ above the non-treated (negative control) mouse sera. The geometric mean titre (GMT) of these starting points was calculated for each treatment group. GraphPad Prism 5 was used for all statistical analyses performed in this study. Chi squared tests were used to determine the statistical significance between means or medians using probabilities of p<0.05 as the limit for observed differences to be considered significant. A two-tailed unpaired t-test was performed to evaluate the statistical difference between two groups of plant or animal data.
